# Restless Legs Syndrome in End Stage Renal Disease Patients on Haemodialysis

**DOI:** 10.12669/pjms.306.5691

**Published:** 2014

**Authors:** Irfan Haider, Muhammad Anees, Syed Adnan Hussain Shahid

**Affiliations:** 1 Dr. Irfan Haider FCPS (General Medicine), Senior Registrar Department of Medicine, Fatima Memorial Hospital, Lahore, Pakistan. Postgraduate Trainee FCPS Nephrology, Mayo Hospital, Lahore, Pakistan.; 2 Dr. Muhammad Anees, FCPS (Nephrology), Assistant Professor Nephrology, Head of the Nephrology Department, King Edward Medical University and Affiliated Hospitals Lahore, Pakistan.; 3 Dr. Syed Adnan Hussain Shahid FCPS (General Medicine), Assistant Professor Medicine, Department of Medicine, Fatima Memorial Hospital, Lahore, Pakistan.

**Keywords:** Restless legs syndrome, End stage renal disease, Haemodialysis

## Abstract

***Objective:*** This study was cross sectional survey conducted to find the prevalence of Restless legs syndrome (RLS) in end stage renal disease (ESRD) patients on haemodialysis (HD).

***Methods:*** Data were obtained from 250 patients on chronic maintenance HD. To assess the prevalence of RLS, Clinical diagnostic criteria for RLS was used which is established by the International RLS Study Group.

***Results:*** Total 250 patients were included in this study. 153 (61.2%) patients were male and 97 (38.8%) were females. Mean age of the patients was 45.27 years. Mean duration of HD was 26.10 months. Total162 (64.8%) patients were found to be suffering from RLS. Out of 153 males, 87(56%) were suffering from RLS and among 97 females, 75(77.3%) were suffering from RLS. In our study, gender was statistically significantly associated with RLS (p-value 0.001). In age groups 159(63.6%) patients were below 51 years; among them 102(64.1%) were suffering from RLS; whereas 91(36.4%) patients were equal or above 51 years of age and among this group 60(65.9%) patients were suffering from RLS. There was no statistically significant association between RLS and age groups (p-value 0.776).

***Conclusions:*** RLS is common in patients undergoing regular HD. It is more commonly seen in females.

## INTRODUCTION

Restless legs syndrome (RLS), was first described in medical literature by K. A. Ekbom in 1944.^1^ Possible factors which can lead to the development of RLS include decrease in dopaminergic modulation of intracortical excitability, with reduced supraspinal inhibition and increased spinal cord excitability.^1^ It is expected that there are around 1.5 million end stage renal disease (ESRD) patients in South Asia. On the basis of health care facilities availability incidence of ESRD is likely to be higher than that reported from the developed world; Jha V did a study in India in which he assessed the age-adjusted incidence of ESRD was 232 cases per million population per year in South Asia.^2^

The prevalence of RLS in general population is estimated in most of the developed countries as 10 to 15%.^3^^,^^4^ In ESRD the prevalence of RLS is much higher than general population; and also there is high degree of variability in the prevalence in different parts of the world the prevalence ranges from 13.3% to 28%.^5^^,^^6^ The proposed possibilities for these large variations are difference in the diagnostic criteria used for the diagnosis of RLS and genetic variations in different populations.

RLS is diagnosed clinically, on the basis of 2012 revised International restless legs study group (IRLGSG) diagnostic criteria.^7^ In ESRD population studies have proven that presence of RLS is associated with poor quality of life and increase in mortality.^8^ RLS patients also suffer from sleep disorders which includes insomnia and excessive day time sleepiness.^8^^,^^9^ In general population RLS in more prevalent in females and old age. Few studies have concluded that there is significant impact of social class, psychological status and diabetes mellitus on frequency of RLS.^10^^,^^11^ Potential causes of RLS in general and ESRD patients include anemia, iron deficiency, parkinsonism, peripheral neuropathy and pregnancy.^1^^,^^10^ Considering only ESRD population, predisposing factors that can lead to RLS may include iron deficiency, anemia and under dialysis.^12^

We performed this study in patients suffering from ESRD on haemodialysis (HD) to find out the frequency of RLS in Pakistani population.

## METHODS

This was a cross sectional survey. This study was conducted in haemodialysis unit of nephrology departments, Fatima Memorial Hospital, Lahore and Mayo hospital Lahore. The data was collected from November 2013 to April 2014. Sampling technique was non-probability: purposive sampling. Sample size of 250 cases was calculated with 95 % confidence level and 5 % margin of error and taking percentage of RLS i.e. 20.3% in patients with end stage renal disease on HD.^13^

Inclusion criteria included Age 14-85 years, Gender Male/Female, Patients suffering from end stage renal disease on HD and Patients who are undergoing twice weekly four hours HD. Exclusion criteria included patients who were pregnant or suffering from parkinson’s disease, myalgia, venous stasis, leg edema, arthritis, leg cramps, positional discomfort and habitual foot tapping.

RLS was diagnosed clinically, on the basis of 2012 revised International restless legs study group (IRLGSG) diagnostic criteria.^7^ This criteria consists of five cardinal symptoms all of which were present in patients who were diagnosed as suffering from RLS this includes: 

(i) The urge to move the legs usually not always accompanied by or felt to be caused by uncomfortable and unpleasant sensations in the legs

(ii) The urge to move the legs and any accompanying unpleasant sensation begin or worsen during periods of rest or inactivity such as lying down or sitting..

(iii) The urge to move the legs and accompanying unpleasant sensations are partially and totally relieved by movements, such as walking and stretching, at least as long as activity continues 

(iv) The urge to move the legs and any accompanying unpleasant sensations during rest or inactivity only occur or are worse in the evening or night than during the day 

(v) The occurrence of the above features are not solely accounted for as symptoms primary to another medical or behavioral condition (e.g., myalgia, venous stasis, leg edema, arthritis, leg cramps, positional discomfort, habitual foot tapping).

During the filling of proforma the examiner was available to clarify any misunderstandings the patient may had about the questions. The examiner only marked the patient's answers to avoid any bias or diagnosis of RLS.

Data was analyzed in SPSS version 20.0. Chi-square test was used to see any significant difference in frequency of RLS among the males and females and below 51 years and equal to or more than 51 years of age groups taking P-value < 0.05 as significant.

## RESULTS

Total 278 patients were approached out of which 250 patients were included in this study; 28 patients were rejected as they refused to disclose their personal information for publication purpose. Total 153 (61.2%) patients were males and 97 (38.8%) were females. Mean age of the patients was 45.27±13.90 years ranging from 14 to 78 years (although in our inclusion criteria age range for inclusion was from 15 to 85 years; whereas while collecting data we found patients from age 14-78 years). Mean duration of HD was 26.10 months ± 30.22 1SD months ranging from one month to 216 months. Total 162(64.8%) patients were found to be suffering from RLS. Out of 153 males, 87(56%) were suffering from RLS and among 97 females, 75(77.3%) were suffering from RLS. In our study, gender was statistically significantly associated with RLS (p-value 0.001). In age groups 159(63.6%) patients were below 51 years; among these 102(64.1%) were suffering from RLS; whereas 91(36.4%) patients were above 51 years of age and among this group 60(65.9%) patients were suffering from RLS. There was no statistically significant association between RLS and age groups (p-value 0.776).

## DISCUSSION

This study was done to determine the frequency of RLS in patients of ESRD on HD. In our study results prove that RLS is remarkably prevalent (frequency 64.4%) in ESRD patients on HD and is more commonly seen in females. In different studies done all over world the frequency of RLS in general population is reported to be between 10 to 15%^3^^,^^4^ and in patients of ESRD on HD is 13.3 to 28%.^5^^,^^6^ In our analysis there are three major reasons for this variation in frequency, first the heterogeneity of the study populations that is genetic variation, second the definitions of RLS and the tools used to diagnose the syndrome third and the most important is the associated factors which could lead to the precipitation of RLS include Iron deficiency, diabetes mellitus, peripheral neuropathy and inadequacy of HD. When we compare our study with other studies that used the IRLSSG criteria,^7^ we still find significant differences. The frequency of RLS was 64.8% in our study, 6% (121 patients included in study) in the Indian population,^14^ 23% (223 patients included in study) in Japanese,^15^ 20.3-50.22% in Saudi population^13^^,^^16^ and 14-21% in Caucasians.^8^^,^^17^ This suggests that heterogeneity of the study populations that is genetic differences may be a reason for the wide variation reported in the frequency of RLS.^18^

Anemia, iron deficiency and serum calcium level have all been linked to RLS; however, more recent studies have failed to confirm these earlier findings.^19^ However, we found females were more frequently suffering from RLS. Several studies have also found females to have a higher frequency of RLS in ESRD patients on HD; this may be related to the secretion of sex hormones following circadian rhythms.^19^ Aging is also believed to be a risk factor for idiopathic RLS.^20^ However, previous studies have shown that this is not always true in RLS patients undergoing dialysis.^21^

Studies have been done to find out the associated risk factors which may have a role in the frequency of RLS which revealed that smoking was associated with RLS whereas consumption of coffee and tea had a negative effect on RLS.^22^ In a study by Gigli GL et al. they made two groups of dialysis patients one group was suffering from RLS and other was not and then they compared the characteristic of both, they found that period of dialysis dependence was significantly lower in the group negative for RLS. The use of drugs did not differ in the two groups, except for lower intake of phosphorus binders and antihypertensive drugs among RLS patients.^23^

Causes of high frequency of RLS in our population are inadequate HD, anemia and malnutrition. Providing adequate dialysis to the patient of ESRD is of utmost importance as inadequate dialysis leads to increased morbidity and mortality.^22^ Adequacy of HD is measured in terms of Kt/V, minimum of 1.2 Kt/V is provided per HD session to the patients whose glomerular filtration rate is < 2ml/min. Minimum three of such adequate HD sessions per week are recommended. Inadequate dialysis (lower levels of Kt/V) was found to be associated with RLS.^22^ In Pakistan most of the dialysis centers are providing twice weekly HD with no measurement of Kt/V value same was true in our case as all our patients were undergoing twice weekly HD which may be the major reason for the highest frequency of RLS in our patients as compared to other studies.

There were few limitations in our study which should be addressed in the future.We did not examine the patients for evidence of diabetic neuropathy, which may contribute to the severity of RLS. However, polyneuropathy only partially explains the increased frequency of RLS in type 2 diabetics.^24^ Iron studies were not done because of the financial restrains. Finally, we did not obtain data on nerve conduction parameters because most of the patients did not agree to undergo such investigations. However, we attempted to exclude other diseases that could mimic RLS by performing a structured interview including peripheral neuropathy, parkinson’s disease, myalgia, venous stasis, leg edema, arthritis, leg cramps, positional discomfort and habitual foot tapping.

**Table-I:** Comparison of different studies on RLS.

**Study**	**Year of publication**	**Country**	**Number of patients**	**Frequency of RLS (%)**
Xiao CG et al.^5^	2012	China	375	13.3%
Salman SM.^13^	2011	Syrian	123	20.3
Araujo SM et al.^17^	2010	Brazil	400	21.5%
Al-Jahdali HH et al.^16^	2009	Saudi Arabia	227	50.22%
Sikandar R et al.^25^	2009	Pakistan	271	30%
Kim JM et al.^6^	2008	Korea	164	28%
Kawauchi A et al.^15^	2006	Japan	228	23%
Mucsi I et al.^8^	2005	Hungary.	333	14%
Siddiqui S et al.^19^	2005	UK	277	45.8%
Our study		Pakistan	250	64.8%

## CONCLUSION

Our study confirms that RLS is common in our patients undergoing regular HD. It is more commonly seen in females. Under dose of HD, poor management of iron deficiency anemia due to financial restraints is major cause for the highest prevalence of RLS in our population. RLS is associated with poor quality of life and increase in mortality. Regular evaluation for the detection of RLS in patients undergoing HD for ESRD is required. Early diagnosis and prompt treatment can improve the quality of life. Further studies are required to search its causative factors associations and treatment strategies.

## Author’s Contributions:

**IH **conceived, designed did data collection, manuscript writing, statistical analysis & editing of manuscript.

**MA, SAHS** did review and final approval of manuscript.

**IH** takes the responsibility and is accountable for all aspects of the work in ensuring that questions related to the accuracy or integrity of any part of the work are appropriately investigated and resolved.

## Notes

***Source of funding:*** Self
